# Capsaicin: Current Understanding of Its Mechanisms and Therapy of Pain and Other Pre-Clinical and Clinical Uses

**DOI:** 10.3390/molecules21070844

**Published:** 2016-06-28

**Authors:** Victor Fattori, Miriam S. N. Hohmann, Ana C. Rossaneis, Felipe A. Pinho-Ribeiro, Waldiceu A. Verri

**Affiliations:** Departamento de Ciências Patológicas, Centro de Ciências Biológicas, Universidade Estadual de Londrina, Rodovia Celso Garcia Cid KM480 PR445, Caixa Postal 10.011, 86057-970 Londrina, Paraná, Brazil; vfattori@outlook.com (V.F.); hohmann.miriam@gmail.com (M.S.N.H.); anacrossaneis@gmail.com (A.C.R.); pinho.fe@gmail.com (F.A.P.-R.)

**Keywords:** analgesia, capsaicinoids, chili peppers, desensitization, TRPV1

## Abstract

In this review, we discuss the importance of capsaicin to the current understanding of neuronal modulation of pain and explore the mechanisms of capsaicin-induced pain. We will focus on the analgesic effects of capsaicin and its clinical applicability in treating pain. Furthermore, we will draw attention to the rationale for other clinical therapeutic uses and implications of capsaicin in diseases such as obesity, diabetes, cardiovascular conditions, cancer, airway diseases, itch, gastric, and urological disorders.

## 1. Introduction

Capsaicin is a compound found in chili peppers and responsible for their burning and irritant effect. In addition to the sensation of heat, capsaicin produces pain and, for this reason, is an important tool in the study of pain. Although our understanding of pain mechanisms has evolved greatly through the development of new techniques, experimental tools are still extremely necessary and widely used. Among these basic experimental tools for the study of pain mechanisms and development of novel analgesics, we can fairly consider capsaicin as one of the most important sources of knowledge in the pain field. Curiously, many recent studies have confirmed scientifically what was already known by some cultures: capsaicin can also be used to relieve pain [1]. This paradox can also be seen with opioids, which have an established clinical use as analgesics, but also induce hyperalgesia [2]. Therefore, the complexities of capsaicin-triggered responses as well as its therapeutic usefulness highlight the importance of understanding its mechanisms of action not only in pain modulation, but also in other pathological conditions. In this review, we will highlight the importance of capsaicin to the current understanding of neuronal modulation of pain and explore some mechanisms of capsaicin-induced pain. We will focus on the analgesic effects of capsaicin and its clinical applicability in treating pain. Furthermore, we will draw attention to the rationale for other clinical therapeutic uses and implications of capsaicin in diseases such as obesity, diabetes, cardiovascular conditions, cancer, airway diseases, itch, gastric, and urological disorders.

### 1.1. Discovery, Natural Sources, Role in Plants, Isolation, and Structure of Capsaicin

Chili peppers contain capsaicin (8-methyl-*N*-vanillyl-6-nonenamide), a phenolic compound responsible for their characteristic taste and pungency. All plants from Capsicum genus produce varied amounts of capsaicin, except *Capsicum annum*, and all of them have been used as a spice ingredient and consumed by humans for over 6000 years [3,4]. The quantities of capsaicin can represent up to 1% of the mass of the chili peppers and, together with salt, represent the most consumed condiment by humans. Capsaicin is an intriguing molecule since the consumption of chili peppers evokes opposing sensations (pleasant and unpleasant) depending on the individual experience and chili pepper consumption habits. The effects of capsaicin go well beyond the taste and its role in plants’ health help us to understand how its use can improve human health [4]. 

The production of capsaicin among plants from the Capsicum genus was well conserved, likely due to its roles in seed germination and protection from parasites. In fact, capsaicin is not equally distributed in all parts of pepper fruit. Its concentration is higher in the area surrounding the seeds (placental tissue) and this localization is related directly to the role of capsaicin in protecting seed germination [5]. The aversion to eating large amounts of capsaicin keeps rodents and other mammals away and this represents an important mechanism to increase the chances of germination since mammals can grind and digest the seeds making them unable to germinate. Birds, on the other hand, cannot feel this unpleasant taste of peppers [6]. Importantly, pepper seeds resist to birds´ digestive tract, making them the perfect consumers. Capsaicin also protects plants from parasites such as insects and mold, and humans have been using this property to treat infectious diseases and to preserve food [7,8].

**Figure 1:**
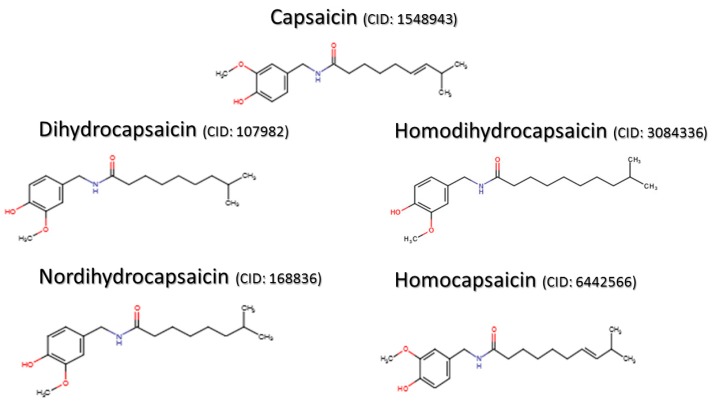
Chemical structure of capsaicin and capsaicinoids. Molecules of capsaicin and capsaicinoids available in PubChem database [12,13,14,15,16]. Compound identifier (CID) number is provided in parentheses. Molecules were drawn using Marvin JS, MarvinSketch in JavaScript.

Despite the unpleasant sensation that occurs when large quantities of chili peppers are consumed, capsaicin promotes pain relief when used in the right dosage and frequency. These properties caught the attention of researchers long ago and still do nowadays, boosting our knowledge about capsaicin. Capsaicin was first purified in 1876 [9] but its structure started to be described only in 1919 [10]. Currently, the structure and properties of capsaicin are well defined (Figure 1). Capsaicin presents a nonpolar phenolic structure and thus cannot be solubilized in water. The main solvents used to extract and maintain capsaicin properties are nonpolar solvents such as ether, benzene, dimethyl sulfoxide and acetone, but ethanol can also be used as a solvent due to its mixed properties.

Because of its chemical structure, capsaicin can be well absorbed when administered topically or orally, reaching up to 94% of absorption [11]. Following its discovery and characterization, it was observed that capsaicin is actually part of a family of compounds that share similar structural and biologic characteristics.

### 1.2. Capsaicin-Derived Molecules and Analogs

Plants from Capsicum genus produce many capsaicin-related compounds. Due to their similarity with capsaicin, these molecules can be grouped in a family called capsaicinoids. Capsaicinoids include dihydrocapsaicin, nordihydrocapsaicin, homodihydrocapsaicin, and homocapsaicin (Figure 1). All these molecules share structural and activity similarities with capsaicin [17,18], but they are not as abundant as capsaicin that can account for up to 80% of capsaicinoid content of chili peppers. The pungency of all these molecules emphasizes the fact that this activity is defined mainly by the benzene ring region, however, the length of acyl chain can modify it [19]. Besides capsaicinoids, there are other groups of molecules that share similarities with capsaicin such as capsinoids, with reduced pungency, and the extremely potent resiniferoids [20,21]. Importantly, all these capsaicin-related molecules present therapeutic properties to treat pain and other conditions and have been used in research to understand the pathophysiology of pain and diseases. Capsaicin has opened the path to our understanding of pain mechanisms and demonstrated that, although counter-intuitive at first sight, it is possible to treat pain by boosting algesic pathways. Furthermore, the ability of capsaicin to cause activity-induced tolerance to pain demonstrates the complexity of a single pharmacological tool that is able either to trigger or treat pathological pain.

## 2. Capsaicin and Pain

Capsaicin selectively stimulates nociceptive neurons and has been widely used to study pain-related events. In this topic, we will highlight some aspects of how capsaicin induces pain and its importance to the current understanding of neuronal mechanisms of pain.

### 2.1. Importance of Capsaicin in Pain Research

Before the discovery of the capsaicin-activated receptor, intradermal injection of capsaicin was used to produce primary and secondary hypersensitivity to noxious and innocuous stimuli in both monkeys and rats [22,23]. Seminal works demonstrated that capsaicin excites nociceptors by increasing the influx of ions, such as calcium, in dorsal root ganglion (DRG) neurons [24,25]. Years later, cloning transient receptor potential cation channel subfamily V member 1 (TRPV1) receptor shed light on the mechanism by which capsaicin induces pain [26]. This work is a landmark in the mechanisms of pain since demonstrated that capsaicin induces pain-like behavior by activation of TRPV1 receptors expressed by nociceptors. At that time, TRPV1 receptors were denominated vanilloid receptor 1 (VR1) [26]. More importantly, this discovery has changed our understanding of pain mechanisms since it demonstrates that a receptor-coupled channel expressed by nociceptors detects environment stimuli resulting in nociceptor depolarization and consequently producing pain. Also, this discovery opened avenues to the development of new drugs since Mendelian disorders in these proteins can produce pain [27]. After that, in vivo evidence demonstrated that mice lacking TRPV1 receptors exhibit reduced thermal noxious response and capsaicin-induced paw licking [28]. Whole patch-clamp technique demonstrated that mice lacking TRPV1 receptors present impaired calcium influx in DRG neurons [28]. Therefore, administration of capsaicin in animals was important to elucidate the function of TRPV1 as well as to aid our knowledge about pain processing and modulation. Therefore, the discovery of TRPV1 was essential to validate capsaicin-induced pain models, which can now be used to study neuronal mechanisms of pain, in addition to testing new TRPV1 antagonists and drugs that target the consequences of TRPV1 activation before clinical trials.

### 2.2. Mechanisms of Capsaicin-Induced Pain

One of the first evidence of a selective action of capsaicin on C-polymodal nociceptors was obtained by the capsaicin-evoked response of C-fibers in the cat saphenous nerve. In addition, injection of capsaicin reduces the thermal threshold in both rats and humans [29]. This seminal work demonstrates that capsaicin selectively acts on C-polymodal nociceptors and the thermodependency of sensory effects on animals and humans [29]. Spinal cord mechanisms of capsaicin-evoked mechanical allodynia depend on G-protein and protein kinases (PKA and PKC) and could be reversed by both G-protein and protein kinase inhibitors. For instance, kinase activity may result in an increase of receptor activity as well as an increase of trafficking and cell-surface expression of molecules [23]. In fact, capsaicin activates PKA and PKC that phosphorylate NMDA receptor subunit NR1 at serine residue 890 and 897, and serine residue 896, respectively, which enhances receptor activity [30,31]. Alongside with this, mitogen-activated protein kinase (MAPK) family has been involved in pain-related states and, indeed, capsaicin administration increases the phosphorylation of p38 MAPK in the periphery and spinal cord dorsal horn [32]. Therefore, inhibition of these kinases has helped to define some of the intracellular mechanisms involved in capsaicin-induced central sensitization. In addition to these kinases, the neuropeptide CGRP is another important component in central sensitization. Capsaicin-induced TRPV1 activation stimulates the release of CGRP in the spinal cord, and intrathecal treatment with CGRP antagonist reduces the development and maintenance of mechanical hyperalgesia and secondary allodynia [33].

Capsaicin-induced pain model was also useful to demonstrate the role of reactive oxygen species (ROS) in central sensitization. Despite their pro-hyperalgesic effect per se [34,35], ROS can also be a source of post-translational modification due to their action on redox-sensitive protein residues such as cysteine and serine [36]. In fact, treatment with the ROS scavenger Tempol (4-hydroxy-2,2,6,6-tetramethylpiperidine 1-oxyl) and PBN (*N-tert*-butylnitrone) reduces the activation of neurons in the dorsal horn as observed by the reduction of electrophysiological activity detected by the number of neuronal spikes [37]. As a consequence of that, there is reduction of primary and secondary hyperalgesia, and reduction of neuron responsiveness induced by capsaicin, suggesting a role of ROS in the maintenance of persistent pain [37]. Keratinocytes are in proximity to nociceptors, which may imply a role for these cells in pain. Using Cre-lox technique to promote expression of TRPV1 in keratinocytes demonstrated that capsaicin stimulates TRPV1-expressing keratinocytes inducing c-fos expression in laminae I and II of the ipsilateral spinal cord dorsal horn, which contributes to evoke acute paw-licking nociceptive behavior [38]. This addresses the interaction between keratinocytes and nociceptors in pain-state.

Capsaicin has helped us to understand the mechanisms related to abdominal pain, a condition inherent of patients with irritable bowel syndrome (IBS). Intracolonic injection of capsaicin induces abdominal mechanical hyperalgesia, and pain-related behaviors such as abdominal licking in a morphine-sensitive manner suggesting its nociceptive nature instead of a normal grooming behavior [39]. IBS patients present abdominal mechanical hyperalgesia and allodynia [40]. Nociceptive fibers present in the colon respond to TRPV1 agonist, and, therefore, highlight these receptors as potential targets for abdominal pain [35]. In fact, TRPV1 co-localizes with substance P and calcitonin gene-related peptide (CGRP) in a model of DSS (dextran sulfate sodium)-induced colitis. Substance P and CGRP are two important neuropeptides in pain signaling that together with TRPV1 mediate visceral pain [41]. This is important considering that TRPV1/CGRP pathway is considered an attractive pharmacological approach to treat visceral pain [42]. In vivo functional magnetic resonance imaging (fMRI) further corroborates the importance of TRPV1 receptors and demonstrates the activity of supraspinal mechanisms in capsaicin-induced pain. Injection of capsaicin in wild-type (WT) rats activates putative pain neural circuit, such as Papez circuit, and the habenular system; and TRPV1 receptor deficiency reduces the activation in these same brain regions in response to capsaicin [43]. This is important since it points out to the supraspinal modulation of TRPV1 in pain. And additionally to these mechanisms, TRPV1 also modulates the emotional component of visceral pain [44]. Modulation of TRPV1/CGRP pathway is important in arthritis as well [45]. In fact, intra-articular injection of CGRP in normal or mono-iodoacetate (MIA)-induced arthritis rats reduces the mechanical threshold and increases percentage of sensitized fibers [46], and treatment with CGRP antagonist reduces CGRP- and MIA-induced sensory neuron firing [46], suggesting that peripheral release of CGRP contributes to inflammation and sensitization of joint nociceptors [45].

**Figure 2:**
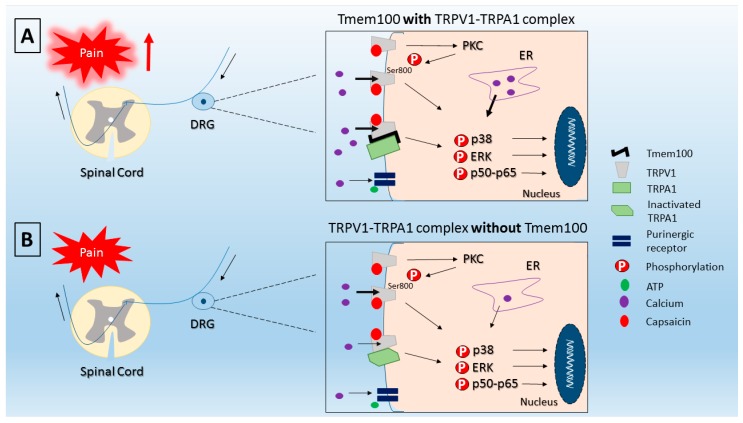
Mechanisms of capsaicin-induced pain. Schematic representation of the phosphorylation at Ser800, which allows TRPV1 discriminating cation influx [50], and participation of Tmem100 in the mechanism of capsaicin-induced pain [49,51,52]. In the presence of Tmem100 (**A**) activation of TRPV1-TRPA1 complex increases the influx of calcium and contributes to higher perception of pain. On the other hand, without Tmem100 (**B**) TRPV1-TRPA1 complex produces lower influx of calcium since TRPA1 is found in an inactivated conformation [49,51,52]. Black thin arrow: lower calcium influx; Black thicker arrow: higher calcium influx; DRG: dorsal root ganglion; ER: endoplasmic reticulum; PKC: protein kinase C.

In the past few years, efforts have been made to identify ligand-receptor and receptor-receptor interactions and their role with pain. Among the first interactions that were shown, we can highlight the capsaicin-TRPV1. In fact, co-administration of capsaicin with QX-314 (a membrane-impermeable sodium channel blocker) facilitates the access of the QX-314 that blocks sodium inward currents in capsaicin-responsive DRG neurons producing analgesia [47]. Nevertheless, in this work, neither the potentially dynamic of TRPV1 permeability to different ions size or charges (unknown at the moment), nor the effect of pore size of the TRPV1 was addressed. TRPV1 receptor was considered a nonselective cation channel with higher affinity for calcium than sodium. TRPV1 agonists such as capsaicin, changes TRPV1 pore size leading to time-dependent discrimination between monovalent and divalent cations over a time frame of seconds that can persist for several minutes [48]. Another striking feature was that phosphorylation of TRPV1 serine 800 residue by PKC allows neurons to discriminate the size of cations by increasing permeability to large cation, and proportionating sensitization of the TRPV1, and enhancement of inward currents [48]. In fact, PKC phosphorylates TRPV1 at serine 800 residues, but not at serine 502, in DRG neurons of rats and contributes to pain in MIA-induced osteoarthritis model [49] (Figure 2). Inhibition of PKC, but not PKA, reduces capsaicin-induced pain-related behavior in MIA-induced osteoarthritis rats [49]. TRPV1 agonists such as N-arachidonoyldopamine (NADA), piperine and resiniferatoxin (RTX) provide distinct pattern of ion selectivity and discrimination [48]. Thus, suggesting that different TRPV1 agonist change the selectivity to inward ions, and the activity of different kinases (such as PKA and PKC) [48,49] could provide different inward ion. Recent data further advanced in this topic by demonstrating that capsaicin binds to TRPV1 pocket as a unique molecule [50].

**Figure 3:**
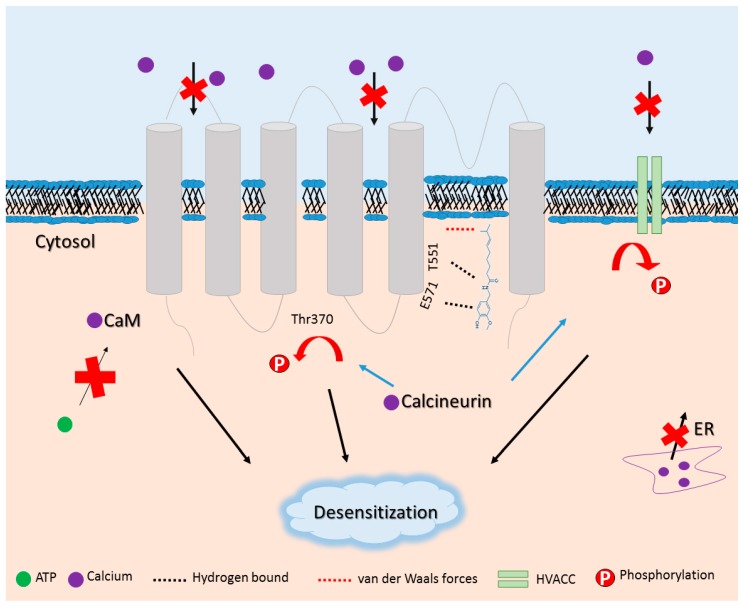
Mechanisms of capsaicin-TRPV1 interaction and desensitization. Capsaicin bounds to TRPV1 in a “’tail-up, head-down configuration” and increases the influx of calcium [50]. A secondary effect due to calcium influx is the activation of calcium-dependent enzymes, such as calcineurin, which dephosphorylates TRPV1 [55,56], downregulates HVACC [57], which culminates in TRPV1 desensitization. Additionally, CaM prevents ATP-induced sensitization of TRPV1 by competing for the same intracellular pocket [55]. CaM: calmodulin; HVACC: high voltage-activated calcium channels; ER: endoplasmic reticulum.

Capsaicin has a very high affinity, sensitivity, and selectivity for TRPV1 and does not activate the homologous TRPV2–TRPV6 receptors [50]. In addition, an elegant work demonstrated how capsaicin binds to TRPV1 and which amino acid residues are involved in this binding. Capsaicin binds to TRPV1 in a “tail-up, head-down configuration” (as coined by the authors). The aliphatic “tail” interacts with the channel through nonspecific van der Waals forces and contributes to binding affinity. Hydrogen bonds between its vanillyl “head” and amide “neck” with residues of glutamic acid E571 and T551 of the channel, respectively, grant specificity for ligand binding [50] (Figure 3). Other interactions with TRPV1, such as Tyr511, Glu570, and Ile569; with the vanillyl “head” allows capsaicin accommodation in this specific pocket (called as vanilloid pocket). On the other hand, RTX (a TRPV1 agonist) molecule is bigger than capsaicin, and possesses a different electron cloud, which does not allow its accommodation in the same vanilloid pocket because this pocket is too shallow for RTX [53]. Therefore, this spatial allocation of both molecules accounts to the distinct agonist pattern and potency explaining the increased potency of RTX compared to capsaicin [53]. In addition to the spatial allocation, structure-activity relationship study demonstrates the functional groups that are essential to these difference. For instance, the amide group is essential for capsaicin activity, while for RTX the five-membered diterpene ring fulfills this role [54]. These studies had an enormous impact because they demonstrated the fundamental pockets to capsaicin or other agonist binding and activation of TRPV1. Therefore, these studies enable future pharmacological approaches based on this knowledge since these agonists can act both as pro-hyperalgesic and anti-hyperalgesic as we will discuss in the next topic.

Regarding receptor-receptor interaction, TRPV1-TRPA1 is a well-documented one [58]. This interaction is attributed to the formation of a heterodimer between TRPV1-TRPA1 receptors [59], which is possible due to lipid raft movement and formation of a cluster of receptors in neurons [60]. Recent evidence demonstrated that a trans-membrane receptor called Tmem100 is co-expressed with both TRPV1-TRPA1 complex in DRG neurons and is essential to modulate their activity by acting as an adaptor molecule [51]. Nevertheless, forming TRPV1-TRPA1 complex without Tmem100 is also possible [51,52]. In the TRPV1-TRPA1 complex without Tmem100, TRPV1 inhibits TRPA1 activity since TRPV1-TRPA1 positive DRG neurons present reduction of inward current after mustard oil (TRPA1 agonist) as stimulus, but not to capsaicin. On the other hand, in the presence of Tmem100 TRPV1 increases TRPA1 activity and potentiates pain perception [51] (Figure 2). Additionally, TRPA1-initiated calcium influx promotes PKA activation, thereby sensitizing TRPV1 channels [61].

Therefore, there is a complex interaction of capsaicin and other agonists with TRPV1 that shed light in the complex pathway to understand TRPV1 modulation. TRPV1 crosstalks with other receptors build up an entirely different pharmacology adding up complexity.

### 2.3. Targeting TRPV1 as a Pharmacological Approach

Currently, capsaicin-induced pain is also used to assess new molecules that target TRPV1 receptor. A whole body of evidence points out to natural product-derived molecules as potential drugs. We recently demonstrated that the flavonoids naringenin [62], vitexin [63], and hesperidin methyl chalcone [64] reduce inflammatory pain by targeting, at least in part, capsaicin-triggered TRPV1 receptors. Other flavonoids also target TRPV1 and reduce pain such as eriodictyol [65] and hesperidin [66], and reduces gastritis such as silymarin [67]. These data corroborate the concept that flavonoids modulate TRPV1. Additionally, other molecules such as α-spinasterol isolated from leaves of the medicinal plant *Vernonia* *tweedieana* (Baker) produce antinociceptive effect by TRPV1 antagonism [68]. Another well-recognized natural product-derived molecule is curcumin, which has more than 100 different targets, among them TRPV1 [69,70]. Curcumin reduces capsaicin-induced calcium rise and inward current in DRG neurons of both mice and rats [69] by antagonizing TRPV1 receptors [71].

Considering the prevalence of chronic pain and the relevance of TRPV1, the pharmaceutical industry has been focusing its efforts in the development of synthetic drugs targeting TRPV1. These drugs are divided into TRPV1 antagonists and TRPV1 agonists [72], and both groups present considerable disadvantages. For instance, TRPV1 agonists can cause pain and/or erythema before desensitization becomes effective, and TRPV1 antagonists usually present lower efficacy compared to TRPV1 agonists and can cause hyperthermia [72,73].

SB-705498 was one of the first developed TRPV1 antagonists. A single oral administration of 400 mg of SB-705498 reduces capsaicin-evoked flare, alongside with elevation of thermal threshold of the patients [74]. As mentioned, hyperthermia is an important side effect due to TRPV1 antagonist administration. In fact, administration of lower doses (2 and 8 mg) of AMG 517 causes hyperthermia that ranges between 39–40.2 °C. On the other hand, repeated administration of this drug for 7 days at a dose of 10 mg reduces hyperthermia, suggesting dose-dependent effect and desensitization [75]. TRPV1 agonists will be discussed in the next section. 

## 3. Mechanisms of Capsaicin-Induced Analgesia 

The effects of capsaicin on nociception are not limited to its ability to produce pain. In fact, high or repeated doses of capsaicin induces an initial pain sensation that is followed by analgesia [76]. This loss of sensitivity to painful stimuli was noticed in response to not only thermal, but also mechanical and chemical noxious stimuli [77]. 

The underlying mechanisms in capsaicin-induced analgesia are being increasingly studied. After exposure to a high or repeated dose of capsaicin, the TRPV1 receptors begin a refractory state commonly termed as desensitization that leads to inhibition of receptor function [78,79,80] (Figure 3). Capsaicin-induced desensitization involves mechanisms not entirely understood. There is evidence that this process includes depletion of neuropeptides such as substance P in the nerve fibers that express TRPV1 [81,82], and an increase of intracellular calcium levels by inhibition of high voltage-activated (HVA) and low-voltage-activated (T-type) calcium channels [83,84,85]. A delayed or secondary effect due to calcium influx is the activation of calcium-dependent proteins that leads to desensitization of TRPV1 [55,56]. For instance, a multi-ligand-binding in the cytosolic ankyrin repeat domain (ARD) of TRPV1 allows intracellular ATP binding to specific pockets of TRPV1-ARD and sensitizes this receptor [55]. On the other hand, desensitization of TRPV1 occurs when calmodulin (CaM) binds in a calcium-dependent manner in the same pockets of ATP, since mutation in these pockets eliminates desensitization in the absence of ATP [55]. Specifically, calcineurin, a CaM and calcium-dependent enzyme, dephosphorylates Thr370 residues that were previously phosphorylated by PKA [56]. Additionally, calcineurin downregulates HVA calcium channels limiting calcium influx in DRG neurons [57] (Figure 3). Altogether, these mechanisms lead to desensitization of TRPV1 and account to capsaicin-induced analgesia.

In addition to the mechanism of TRPV1 desensitization, new evidence has emerged showing the efficacy of capsaicin as an analgesic [86]. Capsaicin activates TRPV1, which inhibits Piezo proteins, a family of mammalian cation-selective ion channels that respond to mechanical stretch [86]. Inhibition of Piezo proteins occurs due to calcium-dependent activation of phospholipase Cδ (PLCδ), which depletes phosphoinositides. In fact, injection of phosphoinositides in the cytosol by excised inside-out patch clamp reduces rundown inward current of Piezo channels and reverts inactivation [86]. Therefore, the depletion of these phosphoinositides correlates with inhibition of mechanical-stimulation of Piezo channels through inhibition of inward current [86]. This work uncovers, at least in part, how local capsaicin produces mechanical analgesia. 

Capsaicin-induced analgesia is also related to degeneration of sensory fibers [87,88,89,90]. The mechanisms through which capsaicin causes cell death are not completely understood. Recent studies indicate that one of the most likely mechanisms is apoptosis via caspase activation. An in vitro study demonstrated capsaicin induces DNA fragmentation and reduction of the nucleus in a caspase-dependent manner secondary to cell death of sensory neurons. In addition, the cell death process triggered by capsaicin via TRPV1 is directly related to mitochondrial permeability transition [91]. On the other hand, capsaicin can promote cell death by apoptosis-independent mechanisms such as cell swelling and bleb formation in the membrane. These mechanisms are dependent on extracellular sodium influx via TRPV1, which in turn is controlled by the intracellular concentration of calcium [92]. Capsaicin-induced analgesia is longer in inflammatory conditions than in basal conditions [93,94]. While the intraplantar injection of 10 µg of capsaicin in control mice produced analgesia for 2 days, in groups stimulated with carrageenan or CFA, the same dose of capsaicin produces analgesic effect for 6 and 30 days, respectively [94]. This enhancement of capsaicin-induced analgesia during inflammation is likely related to a facilitated TRPV1 desensitization [93,94] due to TRPV1 expression [40,95].

**Figure 4:**
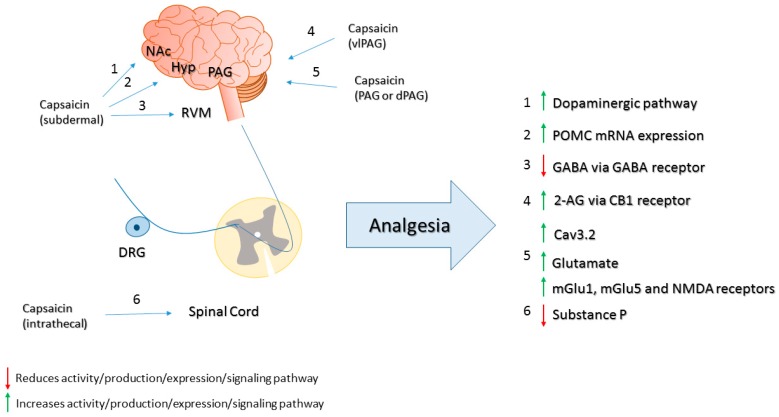
Supraspinal mechanisms of capsaicin-induced analgesia. Subdermal injection of capsaicin produces analgesia by modulating dopaminergic pathway in the NAc (1) [96], opioid pathway in the hippocampus (2) [98], and GABAergic activity in the RVM (3) [96,97]. In addition, vlPAG injection of capsaicin activates endocannabinoid pathway (4) [99], and dPAG by modulating glutamate signaling pathway (5) [100]. Intrathecal injection of capsaicin depletes substance P and also produces analgesia (6) [101,102,103]. DRG: dorsal root ganglion; NAc: nucleus accumbens; Hyp: hippocampus; RVM: rostral ventromedial medulla; PAG: periaqueductal gray; vlPAG: ventrolateral periaqueductal gray; dPAG: dorsal periaqueductal gray;

In addition to peripheral changes, supraspinal mechanisms also modulate capsaicin-induced analgesia. The subdermal injection of capsaicin significantly reduces the jaw-opening reflex and increases the withdrawal threshold to mechanical stimulation in anesthetized rat, and both effects are prevented by microinjection of dopaminergic or opioid antagonist into the nucleus accumbens. The tonic GABAergic inhibition of neurotransmission in the rostral ventromedial medulla (RVM) is also involved in capsaicin-induced analgesia modulation. In agreement, the injection of muscimol (GABA-A receptor agonist), but not naloxone in the RVM prevents capsaicin-induced inhibition of the jaw-opening reflex [96]. This analgesic effect was reversed by intrathecal injection of antagonists of GABA-B and µ-opioid receptors indicating that activation of inhibitory spinal receptors is an important mechanism of capsaicin-induced analgesia [97]. An increase of opioid activity is also observed in the arcuate nucleus of the hypothalamus of rats as assessed by the proopiomelanocortin (POMC) mRNA expression, a precursor of β-endorphin, 20 min after subcutaneous injection of capsaicin [98] (Figure 4).

Capsaicin also induces analgesia when administered centrally in varied foci. For instance, the intrathecal injection of capsaicin or RTX produces long-term regional analgesia with substance P depletion [101,102,103]. The analgesic effect via supraspinal TRPV1 following intracerebroventricular injection of capsaicin depends on the activation of Cav3.2 channels since mice lacking this receptor present higher nociceptive response compared to WT mice [104]. The microinjection of capsaicin in the periaqueductal gray (PAG) [79] or its dorsal portion (dPAG) in rats produces antinociception to thermal stimulation and may be preceded by a short period of hyperalgesia [105]. The analgesic effect of capsaicin in the PAG depends on the release of glutamate and local activation of TRPV1, mGlu1, mGlu5 and NMDA receptors [79]. Additionally, there is a decrease of ON-cell and increase of OFF-cell activation in the RVM [105]. In an animal model of diabetic neuropathy, the injection of capsaicin into the ventrolateral PAG (vlPAG) reduces the thermal hyperalgesia [100]. The injection of capsaicin in the vlPAG leads to the activation of inhibitory descending pain mechanisms. The analgesic effect produced by capsaicin injection in vlPAG depends on local TRPV1 activation that culminates in the release of glutamate into RVM and subsequent activation of OFF-cells and activation of inhibitory descending pain pathway [106]. Additionally, the glutamate released act in mGlu5 post-synaptic receptors leading to Gq-protein-coupled PLCβ-DAGLα pathway-dependent formation of the endocannabinoid 2-arachidonolyglycerol (2-AG). In turn, 2-AG activates pre-synaptic CB_1_ receptors, leading to retrograde disinhibition of GABA release [99]. In addition, there is co-expression of µ-opioid and TRPV1 receptors in vlPAG. Combined administration of capsaicin and µ-opioid receptor agonist sub-doses at this site produces thermal analgesia in rats with increased glutamate release and inhibition of ON-cell activity in RVM [107]. The injection of capsaicin into the RVM inhibits the overt pain-like response in the inflammatory phase of the formalin test in rats with streptozocin-induced diabetic neuropathy, an effect that may be associated with the up-regulation of TRPV1 receptors in the RVM [108] (Figure 4).

Considering the aforementioned evidence, capsaicin has been used as a support pharmacological agent in pain management. Treatment with capsaicin is effective in different types of painful conditions such as complex regional pain syndromes and neuropathic pain [109,110]; postsurgical neuropathic pain [111,112]; post-herpetic neuralgia [113,114] and painful diabetic peripheral neuropathy [115,116]. There is also report that repeated use of nasal capsaicin prevents cluster headache attacks [117]. In humans, topical capsaicin (0.075%) applied four times a day during 3 weeks causes the degeneration of nerve fibers of the skin and consequently decreases sensitivity to cold and tactile stimuli, but to heat and mechanical stimuli [118].

In patients with post-herpetic neuralgia, topical application of 8% capsaicin patch produced a significant decrease in pain for 12 weeks [119,120]. A patient with post-traumatic neuropathic pain presented 80% reduction of the area of allodynia after the use of 8% capsaicin patch. This effect was observed up to the 18th month after application [112]. Oral treatment with capsaicin candy temporarily relieves pain caused by oral mucositis, a common side effect in cancer patients in chemotherapy or radiotherapy treatment [121]. 

The repeated topical application of capsaicin can cause intense burning sensation at both low and high doses. However, the pretreatment with local anesthetic avoids the initial discomfort caused by the use of single high dose of capsaicin [110,122]. The association of local anesthetic lidocaine-derived QX-314 with capsaicin applied in a sensory nerve produces long-lasting analgesia in the orofacial area and inhibits the jaw opening reflex induced by stimulation of the tooth pulp in rats [123]. The perisciatic application of lidocaine (2%) or QX-314 (0.2%) associated with capsaicin (0.05%) in rats after plantar incisional surgery decreases the mechanical hypersensitivity 72 hours after incision and delays the onset of mechanical hypersensitivity by the destruction of TRPV1-expressing afferents. Nevertheless, the delay in the onset of mechanical hypersensitivity was also observed in naïve animals as well as signs of neurotoxicity [124]. The topical association of 3.3% tricyclic antidepressant doxepin and 0.025% capsaicin is able to accelerate the development of analgesia in patients with neuropathic pain compared with the separate use of formulations [125].

## 4. Pre-Clinical and Clinical Uses, and Pharmacological Actions of Capsaicin in Conditions Other than Pain

### 4.1. Capsaicin in Weight Reduction and Obesity

Obesity is an escalating public health challenge globally and a major risk factor for various diseases, including coronary heart disease, hypertension, type 2 diabetes mellitus and cancer [126,127]. Thus, there is urgent need for new therapeutic strategies to treat obesity. In the past decades, numerous studies have shown capsaicin is effective in promoting weight loss and amelioration of obesity [128,129,130]. Herein, we will discuss some of the most relevant mechanisms involved in capsaicin’s anti-obesity effects.

Obesity is the result of an energy imbalance that develops when energy intake exceeds energy expenditure. Capsaicin can limit energy intake while it contains only negligible amounts of energy itself [131,132,133]. Thus, great focus has been turned to studying the effect of capsaicin on energy balance. In humans, the addition of capsaicin to the diet enhances anorexigenic sensations, such as satiety and fullness [132,134]. Moreover, capsaicin decreases ad libitum food intake and suppress orexigenic sensations, i.e., the desire to eat and hunger, in negative and positive energy balance [131,132,135]. Although the exact mechanism of action of capsaicin is not yet fully understood, several plausible mechanisms have been proposed to explain these effects. An early study in rats demonstrated that adding capsaicin in the diet caused an increase in catecholamine secretion in the adrenal medulla via the activation of the central nervous system (CNS) [136,137]. There is an interaction between sympathetic nervous system (SNS) activity and food intake behavior since food intake decreases when SNS activity increases [138]. Therefore, increased SNS activity by capsaicin ingestion suggests that the reduction in energy intake could be due to the anorexigenic effect of catecholamines [133]. Moreover, the consumption of capsaicin increases the concentration of anorexigenic hormone glucagon-like peptide 1 and decreases the concentration of orexigenic hormone ghrelin in humans [139]. Accordingly, oral treatment with capsaicin can regulate high fat diet (HFD)-induced alterations in the expression of several anorectic and orexigenic genes and neuropeptides in the hypothalamus and prevent weight gain in mice [140]. 

Numerous studies have highlighted the role of thermogenesis and increase in energy expenditure (EE) in body weight regulation by capsaicin [130,131,140,141,142,143]. Among potential molecular mechanisms involved in this regulatory effect of capsaicin, activation of TRPV1 appears to be critical as EE is greatly attenuated in mice deficient in TRPV1 and in human individuals having a mutated (Val585Ile) TRPV1 [142]. Increased thermogenesis and EE via capsaicin-induced TRPV1 activation is resultant of catecholamine release and subsequent SNS activation of β-adrenoceptors [143,144]. This mechanism is corroborated by studies showing that the administration of β-adrenergic blockers such as propranolol attenuates thermogenesis [144]. The activation of brown adipose tissue (BAT), which is the major site of sympathetically activated non-shivering thermogenesis, via the TRPV1/β-adrenergic axis, has been shown to be central to the thermogenic effect of capsaicin [142,145]. Nevertheless, other effects such as increased fat mobilization (triglyceride oxidation) in white adipose tissue (WAT) and improved energy metabolism in skeletal muscle mediated by TRPV1 activation also seem to be important in increased EE by capsaicin [142,146]. 

The amount of adipose tissue is tightly regulated and dependent on the differentiation of preadipocytes to adipocytes, a process known as adipogenesis. The modulatory effect of capsaicin on this process has been implicated in the reduction adipose tissue [147,148]. Previous studies have shown that capsaicin reduces the expression of adipocyte differentiation-related proteins PPARγ, C/EBPα, and leptin in a concentration-dependent manner, and the differentiation of 3T3-L1 preadipocytes into adipocytes [149,150,151]. Similarly, capsaicin also inhibits the differentiation of bone marrow mesenchymal stem cells (BMSCs) into adipocytes [152]. Thus, capsaicin-mediated modulation of adipogenesis is not limited to preadipocytes. The inhibitory effect of capsaicin on this process seems to involve the activation of 5’ adenosine monophosphate-activated protein kinase (AMPK) in conjunction with intracellular ROS release [150]. Activated AMPK blocks anabolic pathways and promotes catabolic pathway. Thus, AMPK activation is also linked to inhibition of cell proliferation and apoptosis [153,154]. In support of this concept, capsaicin targets preadipocyte proliferation by blocking the S-phase of the cell cycle [149]. Capsaicin also reduces the number of BMSCs in S phase and induces cell cycle arrest at G0-G1 [152]. Interestingly, capsaicin induces apoptosis in preadipocytes via the activation of caspase-3, Bax, and Bak, cleavage of PARP, and down-regulation of Bcl-2 [151]. Furthermore, capsaicin induces apoptosis in BMSC via increased production of ROS and reactive nitrogen species (RNS) [152]. Thus, the reduction in preadipocyte/adipocyte population and adipose tissue by capsaicin can also be attributed to the inhibition of proliferation and apoptosis.

In addition to the previously discussed mechanisms of capsaicin’s anti-obesity effect, the capsaicin alteration in gut microbial population also seems to be important in preventing HFD-induced weight gain. Oral administration of capsaicin regulated HFD-induced alterations in the abundance of certain bacterial groups in the cecum of Swiss mice, e.g., *Bacterioidetes*, *Firmicutes*, *A. muciniphila*, and *Enterobacteriaceae* [145]. Gut microflora is important in the regulation of host metabolism and energy harvest and may contribute to the development of obesity [155]. In fact, dysbiosis in gut microflora is commonly observed in obese humans and animals [156,157,158]. Therefore, the beneficial alteration in gut microbial population may also be beneficial in HFD-induced obesity.

It is noteworthy that, despite abundant evidence supporting the beneficial role of capsaicin in weight management, some studies have reported no or minimal effects of capsaicin on weight loss in humans [159,160]. Other studies have suggested that the magnitude of capsaicin´s effects on weight loss in humans is actually quite small [131,160]. For instance, 10 kcal negative energy balance, which is the predicted for hedonically acceptable capsaicin doses, in an average weight, middle-aged man would produce an ultimate weight loss of 0.5 kg over 6.5 years [131]. This is important considering that the long-term sustainability is uncertain due to factors such as desensitization upon long-term intake, side effects, and pungency of capsaicin [131,160]. Nevertheless, on a population scale, modest sustained weight loss can be predicted to generate substantial health and economic benefits [161]. Furthermore, it is likely that the analgesic therapy using capsaicin would not reduce the life quality of patients as observed with tricyclic antidepressants, which increase weight gain [162]. Indirectly, the reduction of weight gain will diminish co-morbidities such as knee pain. 

### 4.2. Capsaicin in Glucose Homeostasis and Diabetes

In addition to the effects of capsaicin on body metabolism [130,146], this pungent compound may also have beneficial effects on glucose and insulin homeostasis and diabetes. Dietary and supplementation with capsaicin display an impact on glucose and insulin levels in humans [163,164,165]. Regular consumption of capsaicin-containing chili attenuates postprandial hyperinsulinemia in healthy adults [163] and supplementation with it improves postprandial hyperglycemia and hyperinsulinemia in women with gestational diabetes mellitus (DM) [165]. Further, a crossover study performed on healthy male volunteers revealed that capsaicin lowers glucose and increases insulin levels shortly after oral administration in an oral glucose tolerance test [164]. Importantly, this study not only determined that capsaicin could be detected in the blood as early as 10 min after ingestion and levels maintained for up to 90 min, but also that capsaicin levels correlates with the lower glucose levels and maintenance of the insulin levels [164]. 

Animal studies have reported similar beneficial effects of capsaicin administration on glucose and insulin homeostasis [166,167,168]. Additionally, these studies have also shed light on the mechanisms that may be involved in these effects. For instance, capsaicin may inhibit glucose tolerance by inhibiting adipose tissue inflammatory responses in obesity [169,170]. In vitro, capsaicin suppresses IL-6 and MCP-1 gene expression and protein release from adipose tissue and adipocytes of obese mice [169]. Further, dietary capsaicin markedly reduces adipose tissue macrophages and levels of inflammatory adipocytokines (TNF-α, MCP-1, IL-6, and leptin) and normalizes fasting glucose levels in obese mice [170]. Obesity-related inflammatory proteins can block insulin signaling [171,172]; therefore, capsaicin may reduce glucose tolerance by suppressing their production in obese mice. 

Similarly to many of the other actions described for capsaicin (reviewed herein), there is evidence that the modulation of blood glucose levels and insulin secretion by capsaicin is TRPV1-dependent. Capsaicin induces the secretion of insulin and antihyperglycemic hormone glucagon like peptide-1 in the ileum of WT but not TRPV1^−/−^ mice [173]. Moreover, improved glucose tolerance, insulin levels, and blood glucose profiles by chronic dietary capsaicin are absent in TRPV1^−/−^ mice [173]. In support of this concept, TRPV1 is functionally expressed in islet β-cells, neurons, rat pancreas, and rat β-cell lines RIN and INS1, and capsaicin can modulate insulin secretion by these cells via TRPV1 [167,174,175,176]. In rats, for instance, capsaicin dose-dependently increases insulin secretion and plasma insulin concentrations in TRPV1 expressing islet β-cells and this effect is inhibited by the TRPV1 inhibitor capsazepine [176].

Recent advances in research have revealed that TRPV1 receptors play a central role in the development and progression of type 1 and 2 diabetes [175,177]. In fact, the ablation TRPV1 expressing sensory nerves by capsaicin has been shown to modulate disease development and/or progression [174,175]. Sensory nerves innervating the pancreas are considered major players in the development of pancreatitis and islet inflammation and destruction [174]. Capsaicin-induced permanent elimination of TRPV1-expressing pancreatic sensory neurons reduces islet infiltration, insulin resistance, and β-cell stress in neonatal diabetes-prone non-obese diabetic (NOD) mice [174]. Therefore, capsaicin-induced depletion of TRPV1-expressing neurons prevents the development of diabetes in mice that are genetically predisposed to type 1 diabetes [174]. Similarly, in Zucker diabetic fatty (ZDF) rats, which are used to study various aspects of human type 2 diabetes, the selective elimination of TRPV1 expressing sensory fibers in the islets of Langerhans by capsaicin prevents plasma glucose levels increase and glucose tolerance, and enhances insulin secretion [175]. Interestingly, capsaicin also protects mice from the development of type 1 diabetes via TRPV1 by a mechanism related to gut-mediated immune tolerance. Oral administration of capsaicin attenuates the proliferation and activation of autoreactive T cells in pancreatic lymph nodes (PLNs), protecting mice from diabetes development [177]. The engagement of TRPV1 enhances a discreet population of CD11b^+^/F4/80^+^ macrophages in PLNs, which is essential for capsaicin-mediated attenuation of T-cell proliferation in an IL-10-dependent manner [177]. Therefore, capsaicin/TRPV1 signaling can limit glucose levels increase and diabetes development.

### 4.3. Capsaicin in Cardiovascular Conditions

There is evidence that capsaicin has potential beneficial effects on the cardiovascular system [178,179,180]. The cardiovascular system is rich in capsaicin-sensitive sensory nerves that play a major role in regulating cardiovascular function through the release of neurotransmitters such as CGRP and substance P [180,181]. CGRP is considered to be one of the most powerful vasodilators and plays an important role in regulating blood pressure under both physiological and pathophysiological conditions [182,183,184]. Capsaicin stimulates the release of CGRP through the activation of TRPV1 and therefore decreases blood pressure [180,185]. However, the protective effects of endogenous CGRP rely on the intact function of capsaicin-sensitive sensory nerves since high dose of capsaicin pretreatment, which selectively depletes transmitters in capsaicin-sensitive sensory nerves, could abolish the protective effects of CGRP or even enhance hypertension [186,187,188]. Although blood pressure regulation by capsaicin-stimulated CGRP release is more widely described, dietary capsaicin has also been shown to reduce blood pressure in hypertensive rats and delay the onset of stroke in stroke-prone spontaneously hypertensive rats (SHRsp) by increasing the phosphorylation of PKA and endothelial nitric oxide synthase (eNOS) via TRPV1 activation [189,190]. It is noteworthy to mention that CGRP antagonists, such as Olecegepant (BIBN4096BS), BI44370A, Telcagepant (MK-0970), and MK-3207 do not alter basal blood pressure despite the role of CGRP in regulating blood pressure [191].

In addition to the regulatory effects on blood pressure, other cardioprotective effects have also been described for capsaicin. Long-term activation of TRPV1 by capsaicin decreases lipid storage and atherosclerotic lesions in aortic sinus and thoracoabdominal aorta of mice [192]. Additionally, activation of TRPV1 by capsaicin impedes foam cell formation by inducing autophagy in oxidized low-density lipoprotein (oxLDL)-treated vascular smooth muscle cells and ultimately slows down the process of atherosclerosis [193]. Moreover, it is likely that the antioxidant property of capsaicin also contributes to their protective effects on cardiovascular system. The oxidation of LDL is an initiating factor for the development and progression of atherosclerosis [194]. In vitro, capsaicin increases the resistance of LDL to oxidation by delaying the initiation of oxidation and/or slowing the rate of oxidation [195]. In HFD rats, capsaicin treatment reduces lipid peroxide levels in the serum [196,197]. Moreover, it has been reported that regular consumption of chili for 4 weeks increases the resistance of serum lipoproteins to oxidation in adult men and women [198]. These reports further support the potential clinical value of capsaicin on the prevention of cardiovascular diseases, such as atherosclerosis and coronary heart disease.

Capsaicin has been shown to inhibit platelet aggregation [199,200], which may also provide protection against cardiovascular diseases [201]. Capsaicin’s anti-aggregating effect on platelets is attributed to the alteration in the fluidity of platelet membrane [202,203]. The anti-aggregating effect of capsaicin on platelets seems to be TRPV1-independent since a selective competitive TRPV1 inhibitor A-993610 does not affect the ability of capsaicin to inhibit platelet aggregation [200]. However, there is conflicting data showing TRPV1-dependent pro-aggregating effect of capsaicin, via serotonin release, and adenosine diphosphate- and thrombin-induced platelet activation [204]. Therefore, further investigation is needed to verify the anti-haemostatic property of capsaicin and the mechanisms involved.

### 4.4. Capsaicin in Cancer

Despite several advances in therapies, cancer is still a major cause of morbidity and mortality worldwide [205]. In the past decades, the anticancer activity of capsaicin has been broadly investigated for a variety of cancer types. Capsaicin has been shown to possess chemopreentive and chemotherapeutic effects [206,207], and in vivo studies support the antitumorigenic activity of capsaicin [207,208]. In contrast, there is conflicting evidence that capsaicin may also act as carcinogenic or co-carcinogenic [209], thus capsaicin might play a role in either preventing or causing cancer.

The exact cellular mechanisms involved in capsaicin’s anticancer effects are still not completely understood, however, numerous studies have attributed it to apoptosis, cell-cycle arrest, and anti-angiogenic effects [207,210,211]. Many types of cancer disrupt apoptotic pathways and/or enhance anti-apoptotic ones, and the loss of apoptotic signaling is highly associated with malignancy [212]. Capsaicin can induce apoptosis in over 40 different types of cancer cell lines [213,214]. Some of the mechanisms that have been described are activation of cAMP-activated protein kinase [215] in human osteosarcoma cells and PPARγ-induced apoptosis in HT-29 human colon, endoplasmic reticulum stress in human nasopharyngeal carcinoma and pancreatic cancer cells, down-regulation of STAT3 target genes Bcl2 and survivin in multiple myeloma cells, among others [213]. Interestingly, in many types of cancers, capsaicin exhibits pro-apoptotic activity, which seems to be related to TRPV1 or TRPV6 activation. The activation of these receptors by capsaicin induces calcium-mediated mitochondrial damage and subsequent cytochrome c release [216,217].

Cell cycle and growth arrest are important defense mechanisms against cancer and targets for cancer prevention and therapy [218], and capsaicin has been shown to modulate both. In human bladder cancer cell line 5637, capsaicin induces G0/G1 phase arrest by inhibiting cyclin-dependent kinases (CDK) 2, CDK4 and CDK6 [210]. Similarly, capsaicin reduces in a concentration-dependent manner cyclin D1 in colon cancer cell lines [213,219]. In breast cancer cells, on the other hand, capsaicin induces cell-cycle arrest by modulating the epithelial growth factor receptor/HER2 pathway and p27 expression in estrogen receptor-positive and -negative cells [220]. Taken together, these data show that capsaicin may halt growth and division of cancer cells by targeting cell cycle regulators. Nevertheless, it is important to mention that several other mechanisms of capsaicin-induced cell-cycle arrest have also been described for capsaicin [213].

Angiogenesis is an essential factor for the progression of most types of cancer. It has been demonstrated that capsaicin has anti-angiogenic properties both in vitro and in vivo by interfering with angiogenic signaling pathways [221]. Treatment of endothelial cells with capsaicin suppresses VEGF-induced proliferation, migration and tube formation in mice via down-regulation of p38 MAPK, protein kinase B (PKB or AKT) and focal adhesion kinase (FAK) activation [221]. Further, capsaicin increases the degradation of hypoxia inducible factor 1α in non-small cell lung cancer, which is a key transcription factor for VEGF transcription [222]. Collectively, these studies highlight the anticancer potential of capsaicin by regulating several mechanisms that are commonly altered in cancer cells and are important for tumor growth. 

Despite the mounting evidence supporting a chemo-preventive role for capsaicin in cancer cell culture and animal models, a consensus about whether capsaicin prevents or promotes cancer has not yet been reached [223]. Several animal studies have shown that capsaicin is potentially carcinogenic. For instance, approximately 60% of rats fed a semisynthetic diet containing 10% chilies develop neoplastic changes in the liver [224]. Also, mice fed 0.03% capsaicin in a semisynthetic diet over their lifetime develop benign polypoid adenomas of the cecum [225]. Moreover, studies report that capsaicin may also have co-carcinogenic potential. Topical application of capsaicin on the dorsal skin of mice with 9, 10-dimethylbenz(a)anthracene (DMBA)/12-Otetradecanoylphorbol-13-acetate (a known skin tumor inducer) significantly accelerated tumor formation and growth and induced more and larger skin tumors. Mechanistic study revealed that pre-treatment with capsaicin elevated cyclooxygenase-2 and iNOS and up-regulated the phosphorylation of nuclear factor-kappa B (NF-κB), ERK, and p38, indicating that inflammation, ERK and p38 collectively play a crucial role in cancer-promoting effect of capsaicin in carcinogen-induced skin cancer in mice [226]. Chili extract and hot chili pepper containing capsaicin promoted the development of stomach tumors initiated by methyl-acetoxy methylnitrosamine in mice and increased the incidence of *N*-methyl-*N*-nitrosoguanidine–induced gastric cancer in rats, respectively [227,228]. Furthermore, capsaicin (125 mg/kg)-induced systemic denervation of sensory neurons results in significant increase of lung and cardiac metastases in adult mice injected orthotopically with syngeneic 4T1 mammary carcinoma cells [229]. In line with these findings, many epidemiologic studies indicate that consumption of hot peppers, containing capsaicin, might be associated with an increased risk of cancer, especially gallbladder or gastric cancer [230,231]. However, many of these epidemiologic studies present considerable limitations. 

### 4.5. Capsaicin in Airway Diseases 

Nociceptors play important role in airway diseases such as allergic rhinitis and asthma [232,233], which are accompanied by intense inflammatory infiltrate [232,233,234]. Nociceptors also play an active role in the regulation of immune response since they can recognize and respond to danger and environment stimuli [235,236]. Therefore, the inhibition of their activity in airway diseases may be beneficial to the host [232,233]. In fact, injection of capsaicin in mice exposed to ovalbumin exacerbates airway inflammation by increasing the number of leukocytes in the broncho alveolar lavage fluid (BALF) [232]. Further corroborating this concept, the ablation of the nociceptor by using the Nav1.8-Cre/DTA mice strain [232] or using interference RNA for TRPV1 [233] reduce these same parameters in allergic rhinitis and asthma models, suggesting an endogenous role for TRPV1. In this sense, QX-314 silences nociceptors, which leads to the reduction of the number of infiltrating leukocytes in the BALF, IL-5 production, and improvement of airway inflammation [232]. IL-5 is one of the main cytokines in asthma. In a cascade of events, IL-5 activates in a calcium-dependent manner capsaicin-responsive nodose ganglia and Nav1.8-positive nociceptors, which in turn release vasoactive intestinal polypeptide (VIP). VIP activates innate lymphoid cells 2 (ILC2) and culminates in airway inflammatory exacerbation [232].

Non-allergic rhinitis (NAR) or idiopathic rhinitis (IR) may be described as chronic nasal symptoms, such as obstruction and rhinorrhea that occur in relation to non-allergic, non-infectious triggers such as change in the weather, exposure to caustic odors or cigarette smoke, and barometric pressure differences [237]. Intranasal application of capsaicin has beneficial effects in this type of rhinitis, although this application is initially irritating to the applied area, it can eventually desensitize the sensory neural fibers and reduce nasal hyper-responsiveness [238]. The desensitization of sensory nerves with capsaicin has been shown to provide symptom relief for up to 9 months. Patients treated with intranasal capsaicin reported significantly reduced visual analog scale scores for overall nasal symptoms, rhinorrhea, and nasal blockage [239]. In agreement with previous reports, in a placebo-controlled study with of 24 patients with non-allergic non-infectious perennial rhinitis, the group treated with 0.15 mg capsaicin spray solution over 2 weeks showed significant and long-term reduction in the visual analogue scale scores. However, no significant difference was observed in the concentrations of leukotriene C4, D4 or E4, prostaglandin D2, and tryptase when compared to placebo group [240]. On the other hand, the same dose and treatment protocol used in the previous work showed no significant therapeutic effect in patients with perennial allergic rhinitis due to house dust mite [241], suggesting that the application of capsaicin would be benefit only in non-allergic-related rhinitis. A recent study has shown that NAR/IR is associated with an increased expression of TRPV1 in the nasal mucosa and substance P levels in nasal secretions. Mechanistic studies revealed that capsaicin exerts its therapeutic action by ablating TRPV1-substance P nociceptive signaling pathway in the nasal mucosa [242]. 

The role of capsaicin as a therapeutic agent was not addressed in the work of Talbot et al. [232], therefore, the question whether prolonged administration of capsaicin could produce similar results to those in NAR still remains. In spite of that, this study has shed some light on the role of nociceptors in airway diseases, which highlight these cells as key players in the physiopathology of several diseases. Additionally, this study highlights QX-314 as a solid candidate for the treatment of diseases that TRPV1 plays a role since QX-314 requires an opener (endogenous or exogenous activator of TRPV1) to access nociceptors and inhibit them [47,123,232]. 

### 4.6. Capsaicin in Itch

Itch (pruritus) elicits scratching response, whereas pain causes withdrawal responses. Both itch and pain are detected by primary sensory neurons in DRG and trigeminal ganglion, and therefore, share transduction machinery involving TRPV1, TRPA1, and Toll-like receptors (TLRs) [243]. Despite these similarities, whole population analysis of nociceptors reveals the presence of three distinct populations, which are further divided into seven subgroups. These subgroups are differentiated by the expression of neuronal receptors or ion channels [244]. For instance, DRG neurons of the group VI co-express B-type natriuretic polypeptide b (Nppb) receptor and IL-31ra, which implies these DRG neurons as mediators of itch sensation [244]. This also reveals the highly complex machinery of peripheral nociceptors and uncovers novel receptors as targets for pain or itch relief. In fact, nociceptors play an important role in pruritic diseases [245,246] since silencing nociceptors with QX-314 reduces non-histaminergic and histaminergic itch [245]. Both non-histaminergic and histaminergic itch activate TRPV1 and TRPA1 channels and allow QX-314 entry in DRG neurons [245]. In addition, ablation of nociceptors reduces skin inflammation and psoriatic plaque formation [246]. These set of data highlight specific subsets of nociceptors as important players in itch.

Supporting the role of TRPV1-expressing neurons in itch, treatment with dermal patch of 0.025% of capsaicin reduces itch in psoriatic patients [247,248], although in one of these studies, 18 of 44 patients refer burning, stinging, itching, and redness of the skin [248]. In two other studies, treatment with 8% capsaicin patch reduces itch intensity and frequency in three patients with nostalgia paresthetica [249], and in 7 patients with neuropathic pruritus [250]. Also, in these studies, the majority of the patients referred erythema and moderate pain, pointing out to an important common side effect due to dermal capsaicin treatment. Of note, capsaicin 0.1% reduces allyl isothiocyanate (AITC)-evoked scratching in mice [245]. Regardless of the above mentioned efficacy capsaicin in itch, robust data and further clinical trials are needed to confirm the beneficial properties of capsaicin. In addition, the side effects mentioned can be a drawback to the use of capsaicin in itch. 

### 4.7. Capsaicin in Gastric Disorders

Sensory neurons are responsible for maintenance of gastric integrity [251]. Therefore, the gastroprotective effects of capsaicin lie in the modulation of the sensory neurons, since chemical ablation of these neurons mitigates capsaicin protective effects [252,253,254]. Daily treatment with 400 μg of capsaicin, three times a day, reduces ethanol- and indomethacin-induced gastric mucosal damage in healthy human subjects [255]. In terms of animal models, treatment with capsaicin also reduces indomethacin-induced microbleeding [255]. Corroborating, intragastric administration of capsaicin in rats and dogs attenuates aspirin-, indomethacin- and ethanol-induced gastric damage [251], and enhances gastric protection by stimulating capsaicin-sensitive sensory neurons. This effect was demonstrated using ^51^Cr-EDTA clearance technique, which evaluates epithelial integrity by mucosal blood-to-lumen permeability [254]. The gastroprotective mechanism of capsaicin is due to the activation of TRPV1 at gastric sensory neurons which stimulates the release of CGRP and NO [251,255] since co-treatment of capsaicin and L-nitro-arginine methyl ester (l-NAME, a NOS inhibitor) reduces capsaicin effectiveness in mice [256]. 

*Helicobacter pylori* (H. pylori) is one of the main causative agents of gastric ulcer, and its presence correlates with use of NSAIDs [257]. Capsaicin reduces *H. pylori*-induced gastric ulcer by reducing IL-8 production. In addition, capsaicin also reduces *H. pylori*-induced NF-κB activity evaluated by luciferase activity for p65 subunit and nuclear translocation by confocal immunofluorescence in gastric epithelial cells [258]. Moreover, it is noteworthy that capsaicin per se possess bactericidal activity and inhibits *H. pylori* growth in vitro which may contribute to its protective effect [259]. Thus, the medical premise that consumption of chili peppers may be prejudicial to the host is not entirely true. In fact, epidemiologic studies with 103 patients with peptic ulcer in China [260], and 190 in India [261] suggest that consumption of chili peppers is inversely proportional to the incidence of peptic ulcer pointing out to the gastroprotective effects of capsaicin.

### 4.8. Capsaicin in Urological Disorders

Capsaicin has been studied as an alternative therapy for the relief of the symptoms of neurogenic bladder, a urological disorder that seriously affects the quality of life of patients [262]. Neurogenic bladder is often present in patients with multiple sclerosis, spinal cord injury, and other neurological pathologies. Neurogenic detrusor overactive (NDO) and detrusor hyperreflexia are dysfunctions that characterize neurogenic bladder and lead to urgency and increase in urinary frequency, and in many cases, incontinence [262,263]. Overactive bladder is a clinical condition that resembles neurogenic bladder [264], however, its etiology is not associated with neurological or urogenital diseases [265,266].

The first possibility of clinical use of capsaicin in the treatment of urinary tract disorders was demonstrated using intravesical injection of a 100 mL of 1 mM (30 mg) solution of capsaicin (dissolved in alcohol and saline) in patients with multiple sclerosis that presented bladder detrusor hyperreflexia. The same dose has been used successfully in several studies of patients with neurogenic detrusor over activity after spinal cord injury or neurogenic bladder [267,268,269,270]. The use of alcohol as a solvent can cause irritation and become a limiting factor in the use of capsaicin, causing pelvic pain in more than 50% of patients as reviewed before [271]. The efficacy of an alternative dilution of capsaicin in a glucidic solution to treat patients with neurogenic detrusor over activity was also demonstrated. However, this dilution has not been able to avoid pain reported by treatment with capsaicin [272].

Capsaicin also seems to have a protective effect against bladder disorders. An animal study demonstrated that the pretreatment with capsaicin (125 mg/kg, s.c.) was able to prevent spinal cord injury-induced hyperreflexia of the detrusor in rats. A boost treatment 4-5 days after spinal injury maintained the effect of capsaicin [273].

The effect of capsaicin or RTX is related to the action on TRPV1 receptors in the urinary tract, not only in sensory fibers that innervate these structures, but also in urothelial cells [274,275]. In vitro studies with bladder urothelial cells from non-neurogenic overactive bladder patients showed that expression and activation of TRPV1, as well as capsaicin-sensitivity are increased in comparison with healthy volunteers [276,277]. Capsaicin targeting of TRPV1 receptors in the C-fibers leads to the activation followed by desensitization, being responsible for the beneficial effect of capsaicin on the bladder activity, but also by the initial pain sensation due to their use [262].

The use of both capsaicin and RTX is still not a routine clinical practice and can become an alternative treatment for patients who do not respond to conventional therapy with oral antimuscarinics, especially those with neurogenic bladder. However, both molecules present the disadvantage of repeated intravesical applications and the initial discomfort that may discourage the patient adherence to treatment [262].

## 5. Clinically Available Capsaicin Pharmaceutical Formulations

Among the therapeutic uses of capsaicin in the clinic, the most common is for the management of pain. Low-concentration creams, lotions, and patches containing capsaicin (0.025%–0.1% *wt*/*wt*) intended for daily topical application have been available in most countries since the early 1980s. These topical formulations are usually self-administered medications and often without the requirement of a prescription [278]. Clinical studies have revealed that three to five topical skin applications per day for periods of two to six weeks have modest beneficial effects against various pain syndromes, including post-herpetic neuralgia, diabetic neuropathy, and chronic musculoskeletal pain [279,280]. Another topical capsaicin formulation available is the high concentration patch containing 8% capsaicin, which is widely used to treat post-herpetic neuralgia, HIV neuropathy, and other conditions with neuropathic pain symptoms [281,282]. The capsaicin 8% patch rapidly delivers capsaicin into the skin while minimizing unwanted systemic effects, and it is already approved for treatment of neuropathic pain in Europe and USA (only post-herpetic neuralgia) [116]. Robust clinical data demonstrate the efficacy of 8% patch in the treatment of neuropathic pain [116,283,284]. Of note, in a study with patients with neuropathic pain in Scotland [283], and another involving 629 patients of 22 countries and regions [284], suggest that the 8% patch presents similar efficacy to pregabalin, no differences in time to response between treatments, and therefore, represents a promising alternative for the treatment of neuropathic pain [283,284]. The administration of this formulation requires a single application for 30 or 60 min under the supervision of a health-care professional, which reduces potential variability in administration and a lack of patient compliance, in addition to avoiding environmental exposure of patients to capsaicin [278,281,282]. 

Pharmaceutical formulations for per oral administration of capsaicin are available in the form of capsules containing chili peppers [140]. The therapeutic dose for per oral administration of capsaicin has not been established, however, the generally recommended daily dose stated on labels of commercially available capsules is 1350–4000 mg of capsicum with 0.25% capsaicin. This range of dose has been shown to increase energy expenditure, fat oxidation, thermogenesis, and decrease appetite in humans [130], although both lower (0.4–2 mg) and higher (135–150 mg) doses are also effective in promoting these effects [135,160,285]. Other pharmaceutical formulations containing capsaicin are capsicum nasal sprays and homeopathic preparation of *Capsicum annum* and Eucalyptol nasal sprays. These formulations have been used to treat nonallergic rhinitis and the symptoms associated with this condition [286,287]. Although a therapeutic dose has not been established yet, a previous study has shown that 4 µg/puff of capsicum, three times a day for three consecutive days, is efficacious for non-allergic, non-infectious perennial rhinitis [287].

## 6. Conclusions and Future Perspectives

**Figure 5:**
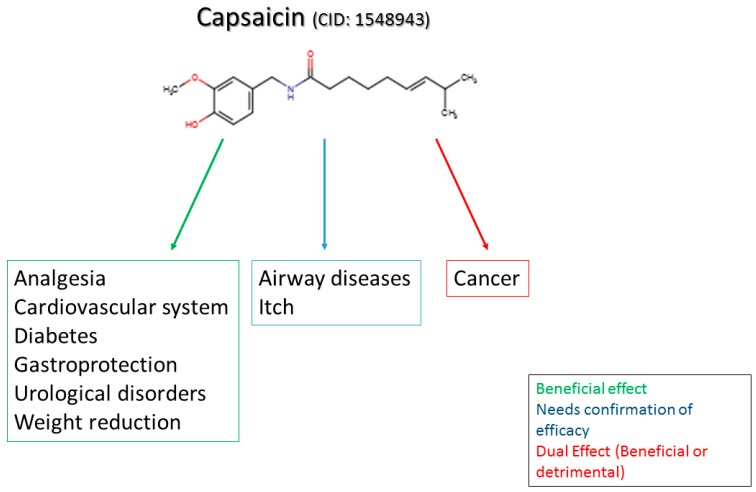
Summary of the current knowledge on capsaicin activities-related to diseases. Green arrow indicates the diseases in which capsaicin presents beneficial effects, and therefore, could be useful as a treatment. Blue arrow indicates diseases in which the effect of capsaicin is still controversial and the therapeutic effect of capsaicin and TRPV1 agonists and antagonists need further investigation. Red arrow indicates that capsaicin might play a role in either preventing or causing cancer.

Capsaicin and food-containing capsaicin have been together with humans over thousands of years, but only more recently that our understanding of how capsaicin affects our organism has significantly advanced. Capsaicin has been essential to our understanding of physiological and pathological processes as well as the relevance of TRPV1 channels. Figure 5 summarizes the pharmacological actions of capsaicin reviewed herein. Capsaicin importance is corroborated by the varied pharmaceutical formulations available and clinical applications, such as the capsaicin 8% patch to treat neuropathic pain. Despite being an old molecule, capsaicin is still a hot topic in scientific community and presents a wide horizon of potential therapeutic uses. Therefore, new pharmaceutical formulations, development of new analogs, or targeting the capsaicin-activated receptor TRPV1 are promising pharmacological approaches in the following years.
